# First insights in terrestrial mammals monitoring in the Candelaria and Machay Reserves in the Ecuadorian Tropical Andes

**DOI:** 10.3897/BDJ.11.e98119

**Published:** 2023-02-27

**Authors:** Juan Pablo Reyes-Puig, Carolina Reyes-Puig, Jessica Pacheco-Esquivel, Santiago Recalde, Fausto Recalde, Darwin Recalde, Jordy Salazar, Eduardo Peña, Silvia Paredes, Marina Robalino, Fernanda Flores, Vladimir Paredes, Edelina Sailema, Gorky Ríos-Alvear

**Affiliations:** 1 Ecominga Foundation, Baños, Ecuador Ecominga Foundation Baños Ecuador; 2 Instituto Nacional de Biodiversidad, Unidad de Investigación, Quito, Ecuador Instituto Nacional de Biodiversidad, Unidad de Investigación Quito Ecuador; 3 Fundación Óscar Efrén Reyes, Baños de Agua Santa, Ecuador Fundación Óscar Efrén Reyes Baños de Agua Santa Ecuador; 4 Universidad San Francisco de Quito, Colegio de Ciencias Biológicas y Ambientales COCIBA, Museo de Zoología & Laboratorio de Zoología Terrestre, Instituto de Biodiversidad Tropical iBIOTROP, Quito, Ecuador Universidad San Francisco de Quito, Colegio de Ciencias Biológicas y Ambientales COCIBA, Museo de Zoología & Laboratorio de Zoología Terrestre, Instituto de Biodiversidad Tropical iBIOTROP Quito Ecuador; 5 Universidad San Francisco de Quito, Instituto BIOSFERA, Quito, Ecuador Universidad San Francisco de Quito, Instituto BIOSFERA Quito Ecuador; 6 WWF, Quito, Ecuador WWF Quito Ecuador; 7 Asociación de Turismo Comunitario Quinde Warmi, Baños - El Placer, Ecuador Asociación de Turismo Comunitario Quinde Warmi Baños - El Placer Ecuador; 8 CIBIO Centro de Investigación em Biodiversidade e Recursos Genéticos, Porto, Portugal CIBIO Centro de Investigación em Biodiversidade e Recursos Genéticos Porto Portugal; 9 Grupo de Biogeografía y Ecología Espacial (BioGeoE2), Facultad de Ciencias de la Vida, Universidad Regional Amazónica Ikiam, Tena, Ecuador Grupo de Biogeografía y Ecología Espacial (BioGeoE2), Facultad de Ciencias de la Vida, Universidad Regional Amazónica Ikiam Tena Ecuador

**Keywords:** ecological corridors, CELS, landscape ecology, mammal diversity, photo identification

## Abstract

Habitat disturbance leads to biodiversity decline and modifications in the landscape structure and composition, affecting both dispersal movements and ecological processes at different temporal and spatial scales. The Ecuadorian Tropical Andes harbour suitable habitats for the distribution of a wide variety of species; however, there is a lack of studies focused on mammal diversity and its association with the habitat attributes in the central-eastern slopes. Here, we reported the diversity of terrestrial mammals recorded between 2019 and 2021 in a camera-trap monitoring study in the Candelaria and Machay reserves in the upper basin of the Pastaza River, Ecuador. We performed site-occupancy probability analysis to assess the influence of spatial variables in the species’ occurrence and also, based on natural marks, we reported preliminary findings in Andean bear individual identification. We detected 22 species of terrestrial mammals. Alpha diversity was similar between reserves with slightly higher species richness in Machay. Evenness indices showed unequal species distribution, with the Andean bear and domestic dogs exhibiting greater dominance. In addition, species composition was dissimilar between reserves, where the species turnover mostly explained the beta diversity. We observed that Andean bear and puma detections increased according to the natural vegetation cover. Conversely, domestic dogs were frequently detected in cells with an increasing proportion of pastures and crops. Additionally, we identified 26 Andean bears and six individuals recaptured during our study. Our results caution about the disturbance derived from human activities since we recorded unprecedented detections of domestic dogs in wild habitats. Nonetheless, it highlights the importance of private conservation areas (e.g. Candelaria, Machay and others) for supporting the occurrence and dispersal of terrestrial mammal species between larger areas in the upper basin of the Pastaza River.

## Introduction

The main causes for the global biodiversity decline include forest clearing, natural resources overexploitation, habitat change, invasive alien species, hunting, climate change, fires and pollution (Pereira et al. 2012, Newbold et al. 2015). Accordingly, land-use change modifies the spatial structure and composition of landscapes and decreases habitat connectivity by reducing suitable habitat patches for wildlife dispersal within the landscape, which ultimately impacts the species’ population viability (Metzger and Décamps 1997, Goodwin and Fahrig 2002, Beier et al. 2011, Lawler et al. 2013, Leonard et al. 2017, Marrotte et al. 2017). However, its effects vary according to the matrix features, the target species and scale of analysis (Krausman 1999, Zeller et al. 2012, Marrotte et al. 2017). For instance, species with broad habitat range take advantage of the interpatch distances and use small area patches as stepping-stones to travel between core areas, whereas species with small habitat range benefit from the small area patches’ contribution to intrapatch connectivity (Goodwin and Fahrig 2002, Herrera et al. 2017). Additionally, habitat loss and fragmentation impact differentially, the former being more negative for connectivity due to its effects on the habitat amount and patch isolation (Goodwin and Fahrig 2002). Similarly, road development is the primary cause of habitat fragmentation and constitutes a barrier to the movement of several species, preventing their dispersal ability to travel and access resources within the landscape (Laurance et al. 2009, Seidler et al. 2015, Beyer et al. 2016, Medrano-Vizcaíno and Espinosa 2021). Thus, increasing habitat connectivity networks (i.e. the availability of routes to support the species' physiological, behavioural and dispersal requirements within the landscape) is a common strategy to improve wildlife conservation planning (Beier and Noss 1998, Belote et al. 2016, Albert et al. 2017).

The tropical Andes are biodiversity hotspots critical for the provision of ecosystem services (Myers et al. 2000, Gaglio et al. 2017). Nonetheless, poor land management and intensive land-use change in anthropogenic landscapes impacts on the species survival rate, either by reducing the habitat availability, species movement capacity or due to the increase in the mortality risk of species occurring close to human features (Nielsen et al. 2010, Cavelier et al. 2011, Fletcher et al. 2019). The foothills and eastern slopes of Ecuador are characterised by rugged topography and dense vegetation that creates montane cloud forests and paramos (Simpson 1975, Sánchez et al. 2018). These ecosystems, typical of the tropical Andes, provide suitable habitats for the distribution of a wide variety of species, many of them with restricted distributions and endemic to the area (Gradstein et al. 2004, Jost and Shepard 2017, Reyes-Puig et al. 2022), but also large and medium-sized mammal species with much larger territory requirements due to their nature and dispersal ability. In this context, studies focused on mammal monitoring on the central-eastern slopes of Ecuador are scarce, as well as those attempting to address the variables affecting the species' site occupancy. Similarly, updated richness and diversity analyses of medium- and large-sized terrestrial mammals through standardised monitoring is not yet available.

In this paper, we present the first results of a two-year pilot camera-trap monitoring study of medium- and large-sized mammals in which we identified the richness and diversity of species of two reserves located in the east-central Andes of Ecuador (Machay and Candelaria), in the upper basin of the Pastaza River. In addition, we estimated the occupancy and detection probability of this group and its association with spatial variables and finally we took advantage of the methodology to recognise unique individuals of spectacled bears that allow us to warn about the importance of the region for habitat connectivity, conservation and management of landscape species.

## Material and methods

### Study area

**Figure 1.**
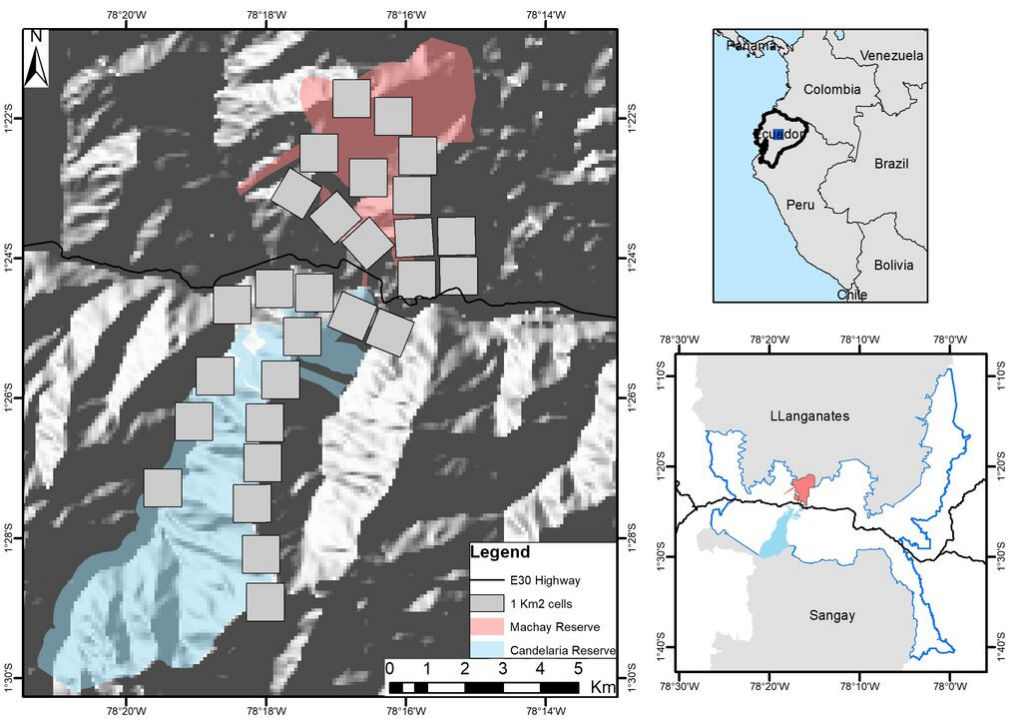
Study area. Large map shows the Machay Reserve in the north and the Candelaria Reserve in the south. Grey squares represent the sampling areas. The bottom right map shows the Llanganates and Sangay National Parks and the study area within the CELS (blue line).

We conducted the study in the eastern slopes of the Andes in Ecuador, in the Cerro Candelaria and Machay Reserves of the Fundación Ecominga (Fig. 1). The study area comprehends 74 km2 (defined as the minimum convex polygon around the camera stations). It is part of the so-called Llanganates – Sangay Ecological corridor (hereafter CELS) which encompasses public and private lands with important remnants of natural habitats between two National Parks, Llanganates to the north and Sangay to the south (Ríos-Alvear and Reyes-Puig 2015). CELS possesses geological and climatic characteristics which have favoured high levels of endemism and species diversification within a narrow territory (Jost 2004). Its strategic location is essential for promoting habitat connectivity (Lessmann et al. 2014, Ríos-Alvear and Reyes-Puig 2015, Cuesta et al. 2017), linking protected areas and well-preserved habitat remnants critical for the conservation of species with different habitat requirements (Reyes Puig and Ríos Alvear 2013, Ríos Alvear and Reyes Puig 2013, Lopez de Vargas Machuca 2014, Reyes-Puig et al. 2015). The study area includes cloud forests and paramos ranging from 1527 to 3710 m in elevation devoted to strict conservation management, embedded in a human-dominated landscape with different degrees of disturbance. The precipitation regime extends from April to July and October to December, with rains up to 5000 mm (Crespo et al. 2011, Ilbay-Yupa et al. 2021). The average annual temperature ranges between 12.3°C and 27.6°C (INAMHI, National Institute of Meteorology and Hydrology- Ecuador 2022).

### Camera trap survey

We conducted a camera-trap survey consisting of four sampling campaigns, from late 2019 to 2021. We defined 15 cells of 1 km^2^ at each Reserve. Each sampling cell was surveyed twice a year with one camera trap active during the dry and rainy seasons. Effective sampling days varied from 12 - 65 due to the camera’s performance and the lockdown and mobility restrictions caused by the COVID-19 pandemic outbreak (Table 1). Sampling cells were selected according to accessibility. We placed one camera per cell at 1 km apart from each other, following wildlife trails to improve species detectability, particularly in areas that potentially favour connectivity between the reserves. Cameras were deployed in the same spot during the entire study. We set a minimum 60-minute interval between each detection (video or photo) as an independent record of the same species (Rovero and Marshall 2009). We defined this range to prevent the bias caused by overcounting the detected species. We used Bushnell Trophy Cam HD Aggressor (model 119875). Cameras were active 24 hours a day, set at normal sensitivity in hybrid mode to record three pictures and one video of 30 seconds per detection event. No attractants were used during sampling.

### Data analysis

#### Species relative abundance

We computed the species relative abundance, based on the average number of independent camera trap records during 100 trap-nights in each Reserve during our study (Rovero and Marshall 2009).

#### Diversity analysis

We estimated alpha and beta diversity for each Reserve. Diversity analyses were conducted following the Hill numbers approach (i.e. the effective number of species) (Jost 2006, Chao et al. 2014). The first four Hill numbers were used: namely H0 as species richness, H1 derived from the Shannon-Wiener exponential index, H2 derived from Simpson’s inverse index and H3 from the Berger-Parker index for species dominance. However, we also included the standard indices from which Hill numbers are derived for comparison. For dominance we applied the Berger-Parker index (d) and for evenness, we used Simpson’s index I, Pielou’s index (J) and Smith & Wilson’s index (Smith and Wilson 1996). In order to compare whether there were significant differences between the alpha diversity of the two localities, we performed a Dunn’s post hoc test. For beta diversity, we took into account the beta partitioning approach, considering the contribution to differences in beta diversity due to species replacement and richness difference (Carvalho et al. 2012, Cardoso et al. 2014). We used Jaccard’s similarity index to assess this pattern. We used interpolation and extrapolation curves to estimate diversity (Chao et al. 2014). For extrapolation, we calculated non-parametric estimators, Chao1 which provide a lower bound on the true diversity of the community, based on abundance data, Chao2 that allows correct sample size using incidence data and Jackknife estimators that reduce the bias for observed richness (Gotelli and Chao 2013). The model used for diversity estimators is derived from sample-based data (incidence data). Here, we did not consider the individuals (abundance data), but the sampling units, which in our case, are camera traps. In this regard, presences are treated as incidences and absences as non-detections for each species within each sampling unit. An incidence matrix is generated and the sum of the rows is used to obtain the incidence-based frequency of a species. Hill numbers are estimated, based on the Bernoulli distribution (Chao et al. 2014b). We performed our analyses with the packages “vegan” (Oksanen et al. 2007), “BAT” (Cardoso et al. 2015), “iNEXT” (Hsieh et al. 2016) and “dunn.test” (Dinno and Dinno 2017) in R.

#### Site-occupancy modelling

We modelled the site-occupancy probability, defined as the proportion of habitat occupied by a particular species under the assumptions of closed population within, but not between seasons, sampling independence and equal probability of occupancy and detection across sampling sites and surveys (MacKenzie et al. 2018). Occupancy models take into account imperfect detection, preventing naïve estimations derived from not detecting the species even when it is present at a site, but also assess the effects of variables as triggers for occupancy and detection probabilities (MacKenzie et al. 2002). We performed multi-season occupancy models to assess the effects of habitat covariates in the occurrence of the terrestrial mammal species in the study area, taking into account the effects of rain seasonality and the presence of domestic dogs as factors leading to changes in occupancy patterns between seasons. We hypothesised the effects of vegetation coverage, protected areas, human accessibility and topography as site attributes affecting occupancy, whereas sampling effort, topography and presence of dogs as sampling variables that influence the probability of detection of wildlife species (MacKenzie and Bailey 2004) (Table 2). We compiled site covariates from Ecuador’s National Thematic Cartography repository and a satellite image classification of a Sentinel 2 image. The site variables were calculated from the location of the camera traps, except the vegetation cover, which corresponds to the average proportion at 1 km^2^ and 250 m of buffer for each camera. Sampling covariates and the presence of dogs were collected from the camera trap records. We estimated site-occupancy probability with the package "unmarked" (Fiske and Chandler 2011).

We assessed the spatial arrangement of detections according to the spatial features within the study area to identify potential characteristics influencing the occurrence of the modelled species. We applied the Wilcoxon-Mann-Whitney test to assess the differences between detections and non-detections within the study area and summarised the results through the “ggstatsplot” R package (Patil 2021).

#### Photo-identification

**Figure 2.**
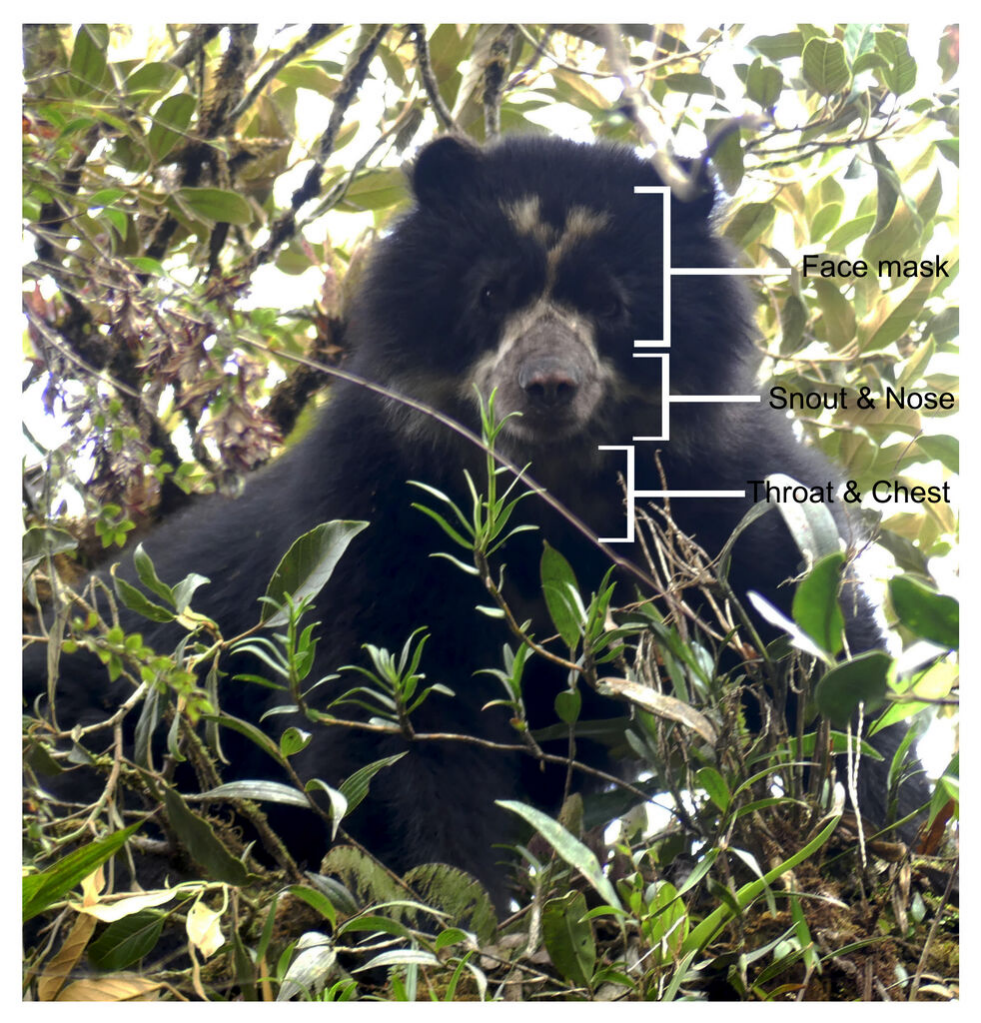
Colouring pattern used for individual identification of Andean bears. Picture by Santiago Recalde.

We performed a photo identification only for Andean bears, based on their facial marks (García-Rangel 2012, Horn et al. 2014) (Fig. 2). We selected the camera-trap records containing Andean bears with facial scenes subject for identification (i.e. clearly visible facial marks). We classified the pictures according to the scene captured (e.g. right-side face, left-side face, front scene, throat) and compared them according to the natural marks photographed. We performed three independent reviews of the photographs between sampling campaigns. In addition, due to the broad home range of the species (Rodriguez et al. 2021), we identified the individuals recaptured between sample campaigns only. We excluded the poor-resolution Andean bear pictures to prevent mistakes and ambiguities in the identification.

## Results

We documented 284 independent camera-trap records of medium and large-size terrestrial mammals in 2814 sampling days at the Candelaria Reserve and 218 records in 2658 sampling days at the Machay Reserve. Species’ relative abundance ranged from 0.03 to 3.13 camera records per 100 sampling days (Table 3).

### Diversity analysis

**Figure 3.**
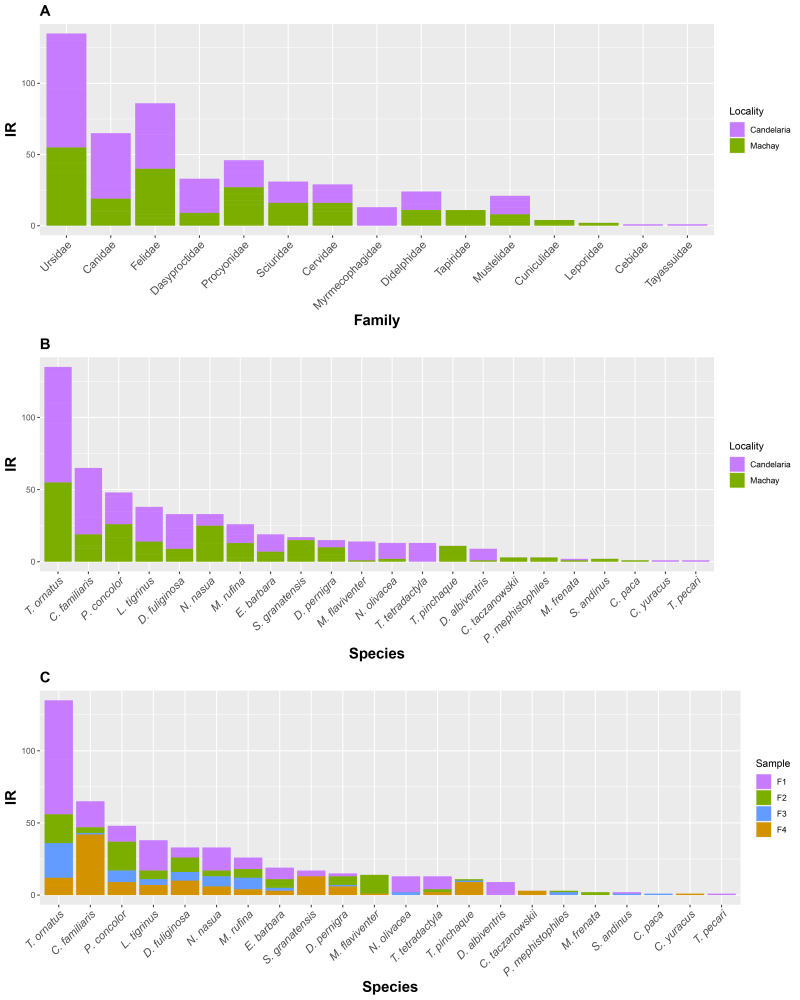
Documented independent records for medium and large-size terrestrial mammal species. **A** By family; **B** By species; **C** By sampling campaign.

We detected a total of 22 species of terrestrial mammals in 502 independent camera-trap records. We recorded 17 species at the Candelaria and 19 at the Machay Reserve. Alpha diversity appears to be similar between Reserves with a slight increase in the number of species in Machay (Table 3). H3 showed some degree of dominant species in the two localities (Table 4). This was corroborated by the total number of independent records detected during sampling campaigns (Fig. 3), where the Andean bear and domestic dogs exhibited the highest number of records. The Pieleu, Simpson and Smith & Wilson Evenness indices showed, as a general trend, that the species relative abundances are not equally distributed, with some degree of variation between sampling campaigns (Table 4). We identified similar values for the Hill numbers in both localities (Table 4). Jaccard Similarity index (β_total_ = 0.64) suggested that the species composition is dissimilar, with some species recorded either in Candelaria or Machay (Suppl. material 1) (Table 3, Fig. 3B). Most of the beta diversity was explained by beta richness difference rather than by beta replacement (β_rep_ = 0.17, β_rich_ = 0.47).

**Figure 4.**
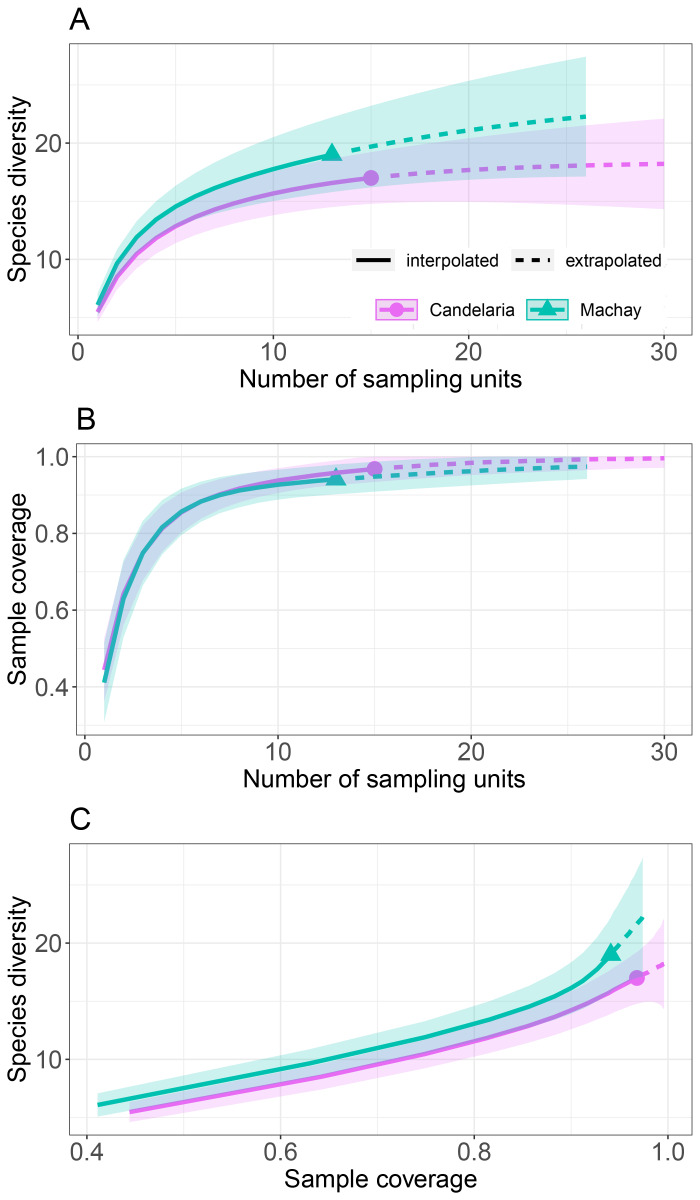
Rarefaction (solid lines) and extrapolation (dashed lines) curves for the medium- and large-size mammal communities in the Candelaria and Machay Reserves. **A** Estimated species diversity according to the sampling units; **B** Estimated sample coverage according to the sampling units; **C** Estimated species diversity according to the sample coverage.

The estimated diversity for Candelaria and Machay communities through rarefaction and extrapolation curves indicated the same trend showed by alpha diversity, identifying Machay as the more diverse locality (Fig. 4, Table 4). The extrapolation to reach the asymptote in species diversity within localities can be reached at 20 sampling units (Fig. 4A). The completeness or sample coverage was high in both locations, reaching the asymptote already at rarefaction (Fig. 4B). First-order Jackknife and Chao estimators were congruent in their estimates, identifying the potential addition of at least two more species in Candelaria and Machay; however, Machay would increase the larger number of species (Table 5).

### Site-occupancy modelling

We estimated the occupancy probability for the Andean bear, puma, oncilla and domestic dogs since they exhibited the highest number of detections recorded during the study (Fig. 3C). Occupancy probability was higher for the Andean bear and puma (Ψ = 0.6 and Ψ = 0.4, respectively), while detection probability was higher for the Andean bear and domestic dogs (p = 0.27 and p = 0.22, respectively) (Table 6). However, we did not find any significant effect of the candidate variables in the occupancy probability for the species modelled (Suppl. material 2).

**Figure 5.**
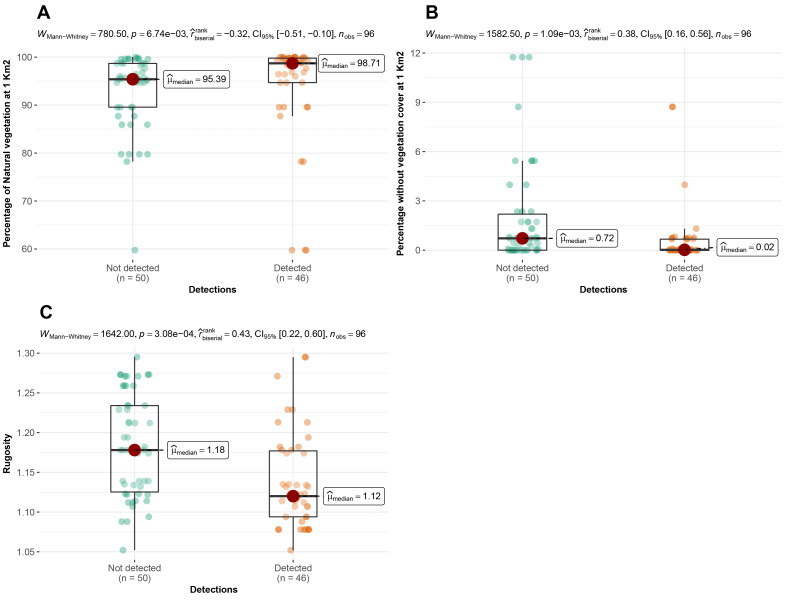
Arrangement of detections of Andean bears according to the spatial variables in the Candelaria and Machay Reserves. **A** Detections according to the percentage of natural vegetation at 1 km^2^; **B** Detections according to the percentage of the area without vegetation at 1 km^2^; **C** Detections according to the rugosity index.

**Figure 6.**
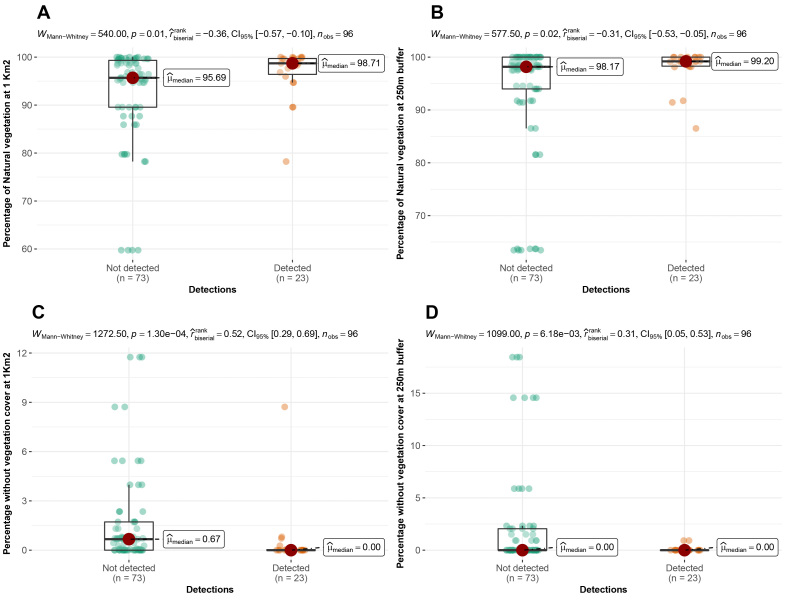
Arrangement of detections of Pumas according to the spatial variables in the Candelaria and Machay Reserves. **A** Detections according to the percentage of natural vegetation at 1 km^2^; **B** Detections according to the percentage of natural vegetation at 250 m buffer; **C** Detections according to the percentage of the area without vegetation at 1 km^2^; **D** Detections according to the percentage of the area without vegetation at 250 m buffer.

**Figure 7.**
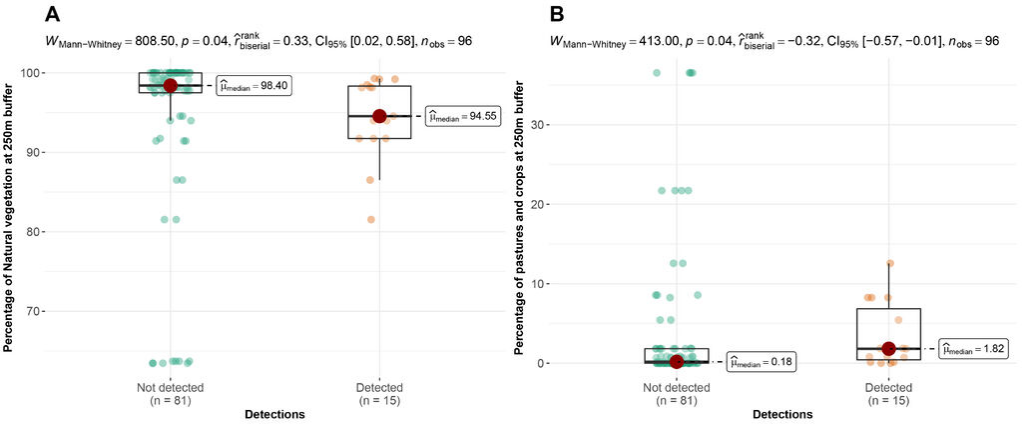
Arrangement of detections of domestic dogs according to the spatial variables in the Candelaria and Machay Reserves. **A** Detections according to the percentage of natural vegetation at 250 m buffer; **B** Detections according to the percentage of pastures and crops at 250 m buffer.

According to the camera traps placement, we observed that 78%, 93% and 74% of Andean bear detections were significantly more frequent in cells with a high proportion of natural vegetation, at very low levels of vegetation absence at a 1 km^2^ scale and in areas with low levels of rugosity, respectively (rugosity index range = 1.05-1.3) (Fig. 5). Pumas were recorded significantly more frequently in cells where the proportion of natural vegetation was high both at 1 km^2^ (78% of detections) and at the buffer scale (87% of detections), but also when the proportion of vegetation absence was low at both scales (Fig. 6). On the contrary, 87% and 73% of domestic dog detections were more frequent in cells with a decreasing proportion of natural vegetation and with an increasing percentage of pastures and crops at the buffer scale (Fig. 7). We did not find any relationship between the detections of Oncilla and the spatial variables evaluated.

### Photo-identification

**Figure 8.**
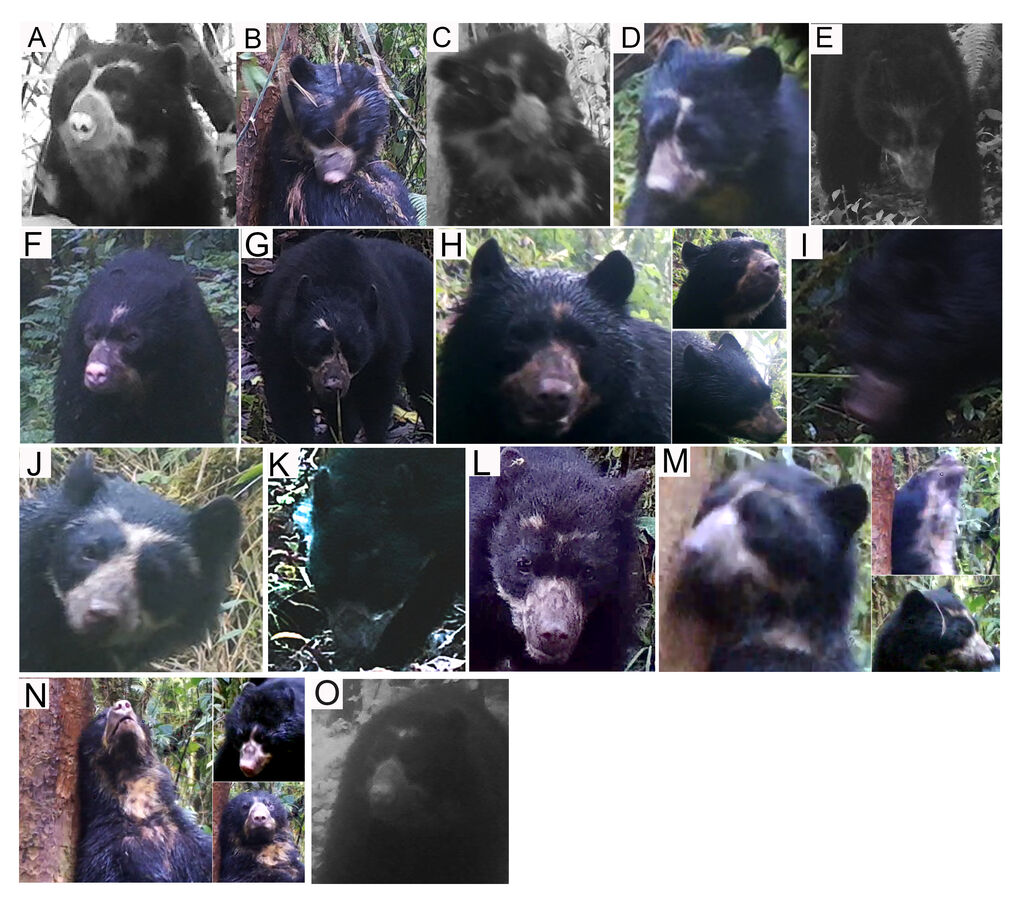
Andean bears identified at the Candelaria Reserve, based on their natural facial marks. Letters correspond to different individuals identified during the study.

**Figure 9.**
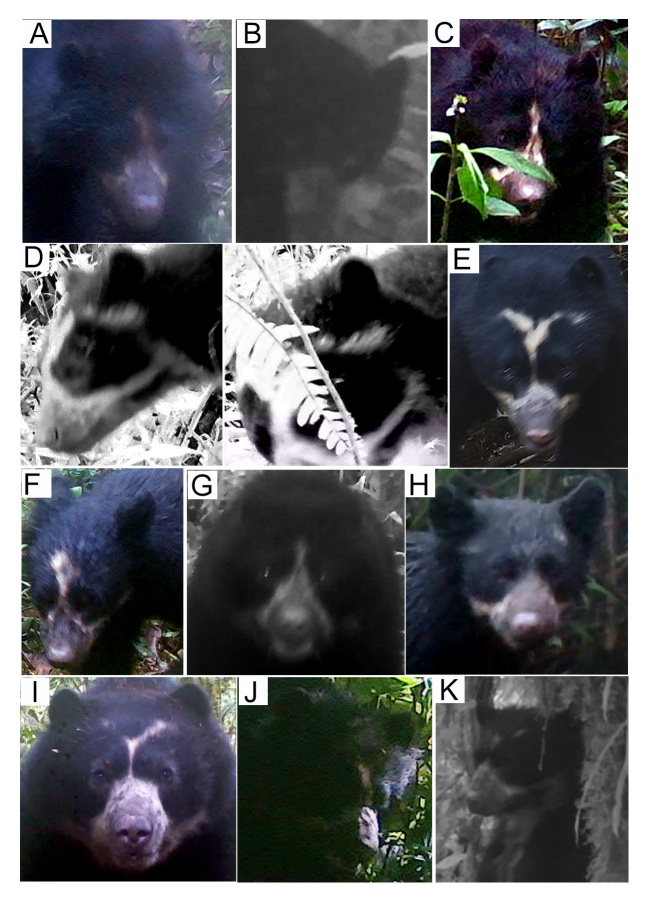
Andean bears identified at the Machay Reserve, based on their natural facial marks. Letters correspond to different individuals identified during the study.

**Figure 10.**
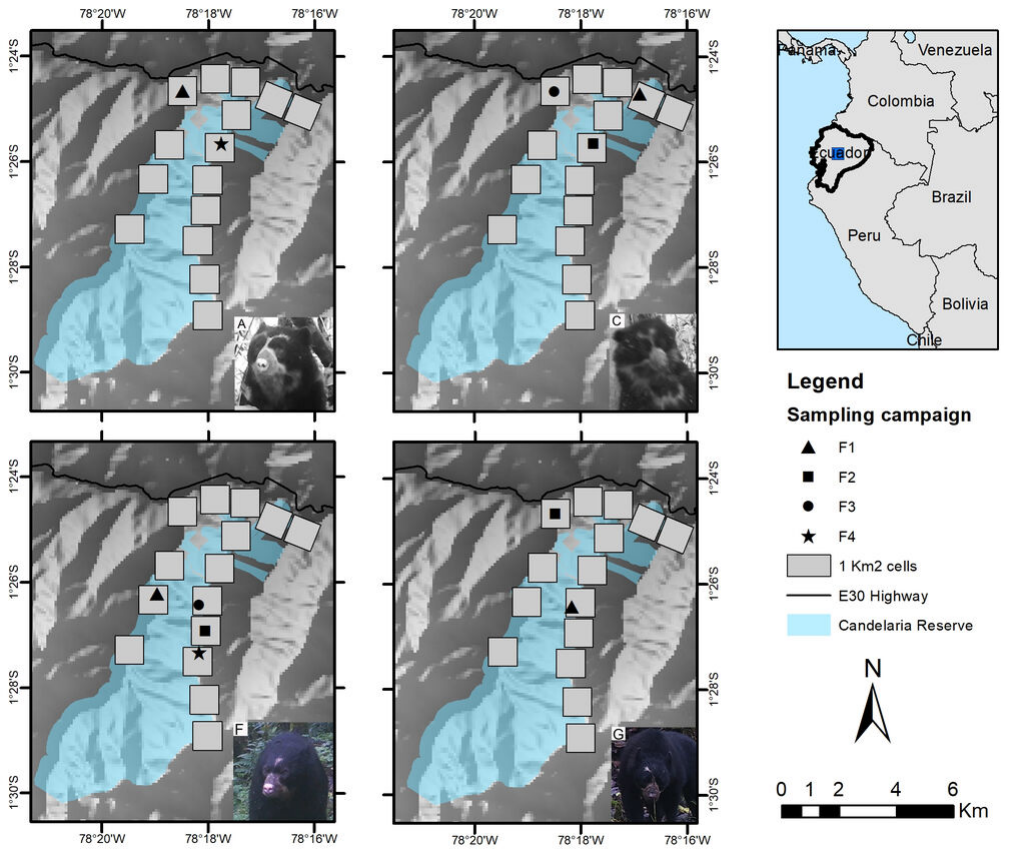
Andean bears recaptured in different sampling campaigns at the Candelaria Reserve.

**Figure 11.**
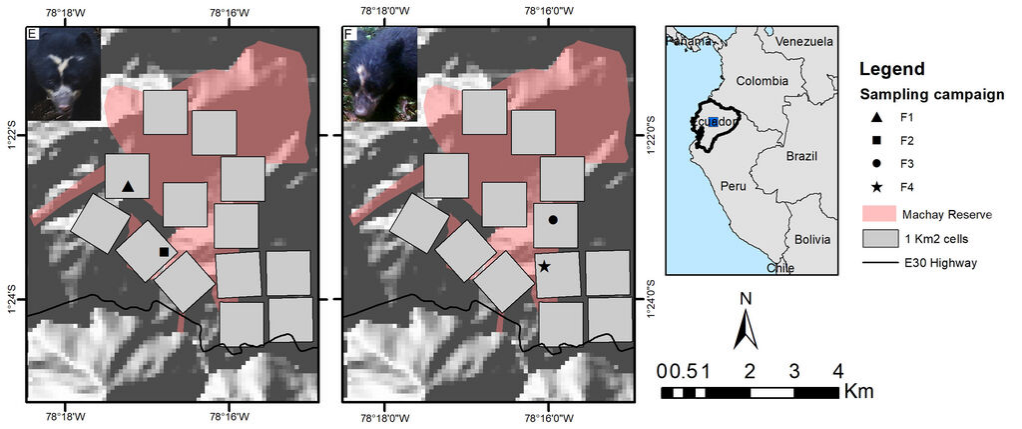
Andean bears recaptured in different sampling campaigns at the Machay Reserve.

We reviewed 756 camera-trap records of Andean bears collected during the study, 72.5% of which correspond to the Candelaria Reserve and 27.5% to the Machay Reserve, comprising 135 independent records. We identified at least 26 Andean bears during the study, 15 in the Candelaria and 11 in the Machay Reserve (Figs 8, 9). According to the sexual dimorphism, marking behaviour and body size, at least 53% of the individuals identified in the Candelaria and 45% in the Machay Reserve corresponded to adult males, while in the latter, only 27% of the individuals identified corresponded to subadults. Nevertheless, we were unable to identify females. In addition, we recorded seven recapture events of four individuals at the Candelaria (Fig. 10) and two recaptures of two individuals at the Machay Reserve between sampling campaigns (Fig. 11). We did not record recaptures of bears between Reserves during the study.

## Discussion

The Andes of Ecuador, Peru and Bolivia are amongst the areas with the highest diversity and endemism of terrestrial mammals in the Neotropics (Ojeda 2013, Noguera-Urbano and Escalante 2015). Our study constitutes the first long-term camera-trap monitoring study conducted in a strategic area that promotes the habitat connectivity between the Llanganates and Sangay National Parks in the eastern slopes of the Andes of Ecuador (i.e. CELS) (Lessmann et al. 2014, Cuesta et al. 2017). Our results reflected that the species richness of terrestrial mammals documented at the Candelaria and Machay Reserves is almost twice that of those recorded in a larger area in the Llanganates National Park (Palacios et al. 2018), although the sampling effort devoted in our study was greater (2320 trap-nights vs. 5232 trap-nights in our study) (Table 3). The diversity analysis showed that the species compositions recorded were moderately different in each Reserve, mainly due to species turnover, which may be a consequence of the species' ecological tolerance (Legendre 2014). For instance, we detected three unique species at the Candelaria Reserve, whereas five species were only detected at the Machay Reserve (Table 3). Our results revealed the dominance of Andean bears and domestic dogs in the study area (Fig. 3C). Accordingly, the relative abundance of Andean bears in our study greatly exceeded those reported by Palacios et al. (2018). We documented unprecedented detections of domestic dogs in the study area (Ríos Alvear and Reyes Puig 2013, Palacios et al. 2018), warning about the increasing human-associated disturbances and the negative interactions between wildlife and dogs (Lenth et al. 2008, Zapata-Ríos and Branch 2016). Similarly, this could have influenced the non-detection of other terrestrial mammals occurring in the study area (e.g. stripped hog-nosed skunk and Andean fox (Palacios et al. 2018)). In this regard, the lack of detections of mountain tapirs in camera traps during our study at the Candelaria Reserve is intriguing. This is not new, since previous camera-trap studies in Candelaria did not detect the species (Ríos Alvear and Reyes Puig 2013), even when there were identified eight different individuals at a 10 km distance in a smaller area and with less sampling effort than the current study (Reyes Puig and Ríos Alvear 2013). According to the species richness expected, we have recorded approximately 73% of the terrestrial mammal species in the area. We expect greater true species richness in the Machay Reserve since 42% of the species recorded are either rare (i.e. singletons and doubletons) or unique species (i.e. species recorded only in one Reserve) (Table 5).

The number of detections conditioned the species occupancy estimation since four of the 22 species documented contained 53% of the total detections during the study, while the 18 species remaining contributed between 0.2 and 6.6% of detections individually (Fig. 3C). Additionally, due to the low sampling size, we were unable to identify covariate effects in the occupancy of the species modelled. However, the null model (i.e. which includes no covariates for detection and occupancy probabilities) revealed that the puma, oncilla and the Andean bear use between 30% and 60% of sampling units in the Candelaria and Machay Reserves, with the Andean bear the species with the highest detection probability (p = 0.27). The occupancy probability estimated for Andean bears in the Candelaria and Machay Reserves is similar to that observed by Springer (2018) in the Chocó-Andean region (Ψ = 0.6 our study vs. Ψ = 0.63) as well as the apparent positive effects of natural forests in the frequency of Andean bear detections within CELS. However, we surveyed a smaller area (78 km2 vs. 804.77 km2) and deployed half the number of camera traps (30 vs. 70 cameras). Our results suggest the occurrence of Andean bears in the Candelaria and Machay Reserves is not affected by the presence of humans and dogs (Springer 2018, Rojas-VeraPinto et al. 2022), since we detected Andean bears close to human-associated features (e.g. roads, pastures and crops) and in camera-traps where we previously documented domestic dogs. However, this must be tested explicitly by, for example, assessing changes in the activity patterns which allows elucidating changes in the bear occurrence as a response to domestic dogs' presence (Zapata-Ríos and Branch 2016, Zapata-Ríos and Branch 2018). Moreover, the high frequency of detections in cells with a low proportion of vegetation absence may reflect the species’ preference for areas that provide natural cover (García-Rangel 2012). However, inhabitants of El Placer, near the Candelaria Reserve, have observed Andean bears roaming through abandoned pastures and feeding on naranjilla crops (*Solanumquitoense*). In addition, we observed more detections in low rugosity areas, opposite to that reported by Rojas-VeraPinto et al. (2022), possibly due to the species exploiting crops for feeding resources (García-Rangel 2012). Detections of pumas were common in cells with almost complete natural vegetation cover and without open areas, suggesting the species evade human disturbance, opposite to what was observed by Figel et al. (2021). On the contrary, our observations suggest that domestic dogs roam in natural habitats close to areas associated with human presence (e.g. pastures and crops) (Paschoal et al. 2018, Zapata-Ríos and Branch 2018). Thus, even though we did not identify explicit effects of the presence of domestic dogs on the probability of pumas occupancy, the spatial arrangement of puma detections does not coincide with the areas where domestic dogs were more frequently detected, suggesting puma's avoidance behaviour of dogs (Zapata-Ríos and Branch 2018).

**Figure 12.**
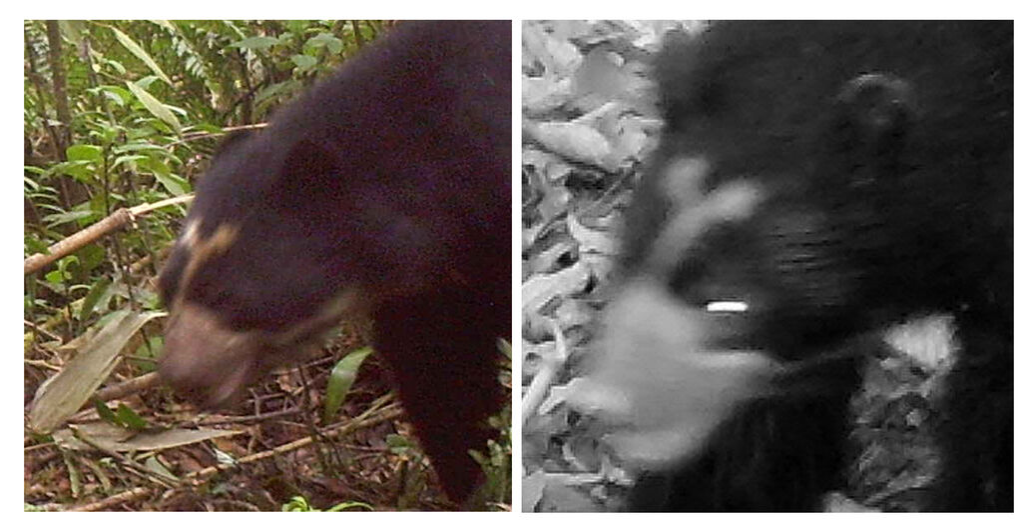
Recapture of an Andean bear recorded in 2011 at the Chamanapamba Natural Reserve (Ríos Alvear and Reyes Puig 2013) (**left**) and the individual C recorded at the Candelaria Reserve in our study (**right**).

The number of Andean bears identified in our study exceeds that documented by Molina et al. (2017) in the Chocó-Andean region and doubles that observed in a previous study at the Candelaria Reserve (Ríos Alvear and Reyes Puig 2013), although our sampling effort was greater (1224, 1050 and 5232 trap-nights, respectively). We are convinced that the true number of bears in the study area is larger than the observed number since we excluded the poor-resolution camera trap records of Andean bears to prevent uncertainty in the photo identification. We recaptured six Andean bears during our study, both in consecutive sampling campaigns and throughout the entire fieldwork (Figs 10, 11). In addition, we suspect the individual C, recorded at the Candelaria Reserve, was previously detected in a survey at the Chamanapamba Natural Reserve in 2011, 10 km to the west of our study area (Ríos Alvear and Reyes Puig 2013) (Fig. 12). To our knowledge, this record depicts the oldest recapture of an Andean bear after eleven years. Due to the individuals identified, our study suggests that Candelaria, Machay and Chamanapamba Reserves, as well as many other natural vegetation remnants within CELS, support the occurrence of landscape species (e.g. Andean bear) and would potentially contribute to the wildlife dispersal between protected areas (Sanderson et al. 2002, Crespo-Gascón and Guerrero-Casado 2019). Additionally, these areas are important refuges of suitable habitats that provide resting and foraging areas for terrestrial mammals within CELS and are critical for managing the habitat connectivity between the Llanganates and Sangay National Parks (Reyes Puig and Ríos Alvear 2013, Ríos Alvear and Reyes Puig 2013, Lessmann et al. 2014, Reyes-Puig et al. 2015, Ríos-Alvear and Reyes-Puig 2015, Cuesta et al. 2017).

Our study highlights the importance of the Candelaria and Machay Reserves as highly diverse areas encroaching in a human-dominated landscape. We expect our results serve as a starting point for establishing a participative landscape scale monitoring network promoting the involvement of conservationists and private stakeholders in CELS. We expect our results to contribute to strengthening the management capacity in the Llanganates and Sangay National Parks, allowing park managers to capitalise on conservation outcomes through coordinated work with local conservationists. In addition, we expect local governments to take advantage of our information to make informed decisions regarding the land-use change and management taking into account the importance of private reserves for strenghthening the habitat connectivity and supporting the endeavour of local conservationists within CELS.

## Supplementary Material

4E80716E-FC05-5FC5-8229-1BCDF27601F3 10.3897/BDJ.11.e98119.suppl1 Supplementary material 1 SM1 Data type Beta diversity coefficients Brief description Accumulated beta diversity considering the beta diversity partitioning approach. File: oo_771112.docx https://binary.pensoft.net/file/771112 Carolina Reyes-Puig, Gorky Ríos Alvear, and Juan Pablo Reyes Puig

1233E256-B554-5FAA-98C3-F9E0666ADD03 10.3897/BDJ.11.e98119.suppl2 Supplementary material 2 Detailed outcomes of the occupancy models assessed. Data type Occupancy modelling outcomes File: oo_812158.docx https://binary.pensoft.net/file/812158 Gorky Ríos-Alvear

## Figures and Tables

**Table 1.** Sampling effort.

**Sampling campaign**	**Date**		**Active cameras**		**Effective sampling days** **Mean (± SD)**	
	**Start**	**End**	**Cerro Candelaria**	**Machay**	**Cerro Candelaria**	**Machay**
**F1**	December 2019	April 2020	12	9	58.66 (± 37.7)	58.3 (± 60)
**F2**	October 2020	December 2020	14	12	49 (± 17.4)	36.2 (± 25.2)
**F3**	April 2021	July 2021	9	9	34.1 (± 34.8)	36.6 (± 33.7)
**F4**	August 2021	October 2021	14	11	45.87(± 29)	46.1(± 31.8)

**Table 2.** Hypothesis for site-occupancy probability estimates. ψ (Occupancy probability) and p (Detection probability) ɣ (Colonisation probability), ε (extinction probability). For j = sites, i = species and K_j_ = surveys at each site.

**Variable**	**Model**	**Description**	**Conceptual model**
**Vegetation (Veg)**	*logit* (ψ_i_)= β*_0_* + *mean* Veg_j_	Proportion of vegetation within the 1 km^2^ sampling cell and at 250 m buffer surrounding each camera trap. We defined three classes: 1. Natural vegetation 2. Pastures and crops 3. Areas without vegetation.	1. The occupancy of wildlife species increases at high proportions of natural vegetation. 2. The occupancy of wildlife species increases at low proportions of pastures and crops. 3. The occupancy increases at low proportions of areas without vegetation.
**Distance to Protected Areas (PAdist)**	*logit* (ψ_i_)= β*_0_* + PAdist_j_	The nearest Euclidean distance from the camera trap to the border of the protected areas.	The occupancy of wildlife species increases closer to the protected areas.
**Distance to creeks (Cdist)**	*logit* (ψ_i_)= β*_0_* + C_j_	The nearest Euclidean distance from the camera trap to creeks.	The occupancy of wildlife species increases closer to creeks as they provide natural corridors for dispersal.
**Distance to roads (Rdist)**	*logit* (ψ_i_)= β*_0_* + Rdist_j_	The nearest Euclidean distance from the camera trap to roads.	The occupancy of wildlife species increases at a high distance from roads.
**Human accessibility (Acces)**	*logit* (ψ_i_)= β*_0_* + Acces_j_	Probability of human access to a pixel as a function of the existence of roads, vicinity to human settlements and navigable rivers (Ríos-Franco et al. 2013).	The occupancy probability increases at a low probability of human accessibility.
**Rugosity (Rugos)**	*logit* (ψ_i_)= β*_0_* + Rugos_j_ *logit* (ψ_i_)= α*_0_* + Rugos_j_	Raster obtained from the MODIS satellite computed as a function of elevation, shape and topographic slope.	The occupancy probability increases at high levels of rugosity as a proxy of less disturbed areas. The detection probability decreases as rugosity increases.
**Forest loss (Floss)**	*logit* (ψ_i_)= β*_0_* + Floss_j_	Rate of tree canopy removal from 2000 - 2018 (RAISG 2020).	The occupancy probability is higher in areas with low rates of forest loss.
**Forest gain (Fgain)**	*logit* (ψ_i_)= β*_0_* + Fgain_j_	The establishment of tree canopy from a non-forest state from 2000 – 2012 (Hansen et al. 2013).	The occupancy probability increases in areas with high rates of forest gain.
**Ecological integrity index (EII)**	*logit* (ψ_i_)= β*_0_* + EII_j_	Forest condition determined by the degree of human pressures and loss of habitat connectivity (Grantham et al. 2020)	The occupancy probability increases in areas with high ecological integrity.
**Rain seasonality (Rain)**	*logit* (ɣ_i_)= β*_0_* + Rain_j_ *logit* (ε_i_)= β*_0_*+ Rain_j_	Indicator variable to reflect the rain regime during the sampling campaign (Crespo et al. 2011, Ilbay-Yupa et al. 2021)	The occupancy changes between sampling seasons according to the rain regime.
**Occurrence of domestic dogs (Dogs)**	*logit* (ψ_i_)= β*_0_* + Dogs_j_ *logit* (ψ_i_)= α*_0_* + Dogs_j_ *logit* (ɣ_i_)= β*_0_*+ Dogs_j_ *logit* (ε_i_)= β*_0_* + Dogs_j_	Occurrence of domestic dogs recorded by the camera traps during the sampling campaign	The occupancy and detection probabilities are higher in areas where domestic dogs are absent. The occurrence of domestic dogs leads to changes in occupancy between seasons.
**Sampling effort (eff)**	*logit* (*z_i_ p*) = α_*0*_ + eff K_j_)	Effective sampling days of the camera traps	Detection probability increases as more sampling effort devoted.

**Table 3.** Photographic rate of the species recorded in the camera trapping monitoring at the Candelaria and Machay Reserves during 2019 – 2021. We compared our findings with a previous study in the Llanganates National Park.

			**Relative abundance (± 90% CI)**				
			**Our study**			**Palacios et al. (2018)**	
**Order**	**Family**	**Species**	**Candelaria**	**Machay**			
Artiodactyla	Cervidae	*Mazamarufina*	0.33 (0.38)	0.62 (0.95)			0.82 (0.81)
		*Pudumephistophiles*		0.16 (0.25)			0.69 (0.70)
	Tayassuidae	*Tayassupecari*	0.04 (0.09)				
Didelphimorphia	Didelphidae	*Didelphisalbiventris*	0.3 (0.7)	0.1 (0.23)			
		*Didelphispernigra*	0.15 (0.28)	0.46 (0.4)			0.39 (0.35)
Carnivora	Canidae	*Canisfamiliaris*	1.42 (1.61)	0.76 (0.6)			
	Felidae	*Leopardustigrinus*	0.76 (0.77)	0.68 (0.43)			0.30 (0.26)
		*Pumaconcolor*	0.8 (0.43)	0.99 (1.2)			0.65 (0.35)
	Mustelidae	*Eirabarbara*	0.33 (0.3)	0.37 (0.33)			
		*Mustelafrenata*	0.03 (0.08)	0.17 (0.41)			
	Procyonidae	*Nasuanasua*	0.27 (0.27)	0.74 (0.26)			
		*Nasuaolivacea*	0.24 (0.46)	0.07 (0.1)			0.26 (0.22)
	Ursidae	*Tremarctosornatus*	3.13 (1.58)	2.32 (3.24)			0.86 (0.48)
Perissodactyla	Tapiridae	*Tapiruspinchaque*		0.4 (0.68)			1.64 (1.22)
Pilosa	Myrmecophagidae	*Tamanduatetradactyla*	0.3 (0.36)				
Primates	Cebidae	*Cebusyuracus*	0.03 (0.06)				
Rodentia	Cuniculidae	*Cuniculuspaca*		0.04 (0.09)			
		*Cuniculustaczanowskii*		0.1 (0.25)			0.34 (0.28)
	Dasyproctidae	*Dasyproctafuliginosa*	0.67 (0.71)	0.4 (0.56)			
	Leporidae	*Sylvilagusandinus*		0.07 (0.16)			
	Sciuridae	*Microsciurusflaviventer*	0.41 (0.97)	0.03 (0.08)			
		*Syntheosciurusgranatensis*	0.05 (0.06)	0.44 (0.88)			

**Table 4.** Summary of medium- and large-size mammal’s diversity indices and Hill numbers. H0: species richness, H1: Shannon-Wiener exponential, H2: the reciprocal of Simpson and H3: Berger-Parker index, J: Pielou’s index, E: Simpson's index.

**Index**	**Candelaria**					**Machay**				
	**F1**	**F2**	**F3**	**F4**	**Total**	**F1**	**F2**	**F3**	**F4**	**Total**
**H0 (S)**	13	12	7	12	17	13	11	14	12	19
**H1 (exp H')**	8.62	9.23	3.65	4.76	10.15	6.34	6.04	10.69	10.31	11.31
**H2 (1/D))**	6.12	7.76	2.47	2.86	7.28	3.91	3.88	8.69	9.29	8.40
**H3 (d)**	4.85	6.93	2.09	2.37	5.90	3.14	3.15	7.63	7.61	6.90
**J**	0.83	0.89	0.67	0.63	0.82	0.72	0.74	0.89	0.94	0.82
**E**	0.47	0.65	0.35	0.24	0.43	0.30	0.35	0.62	0.77	0.44
**Smith & Wilson index**	0.60	0.65	0.76	0.42	0.40	0.64	0.61	0.69	0.71	0.40

**Table 5.** Diversity estimators of medium- and large-size mammals in the Candelaria and Machay Reserves. Obs: Observed species richness, S1: singletones, S2: doubletones, Jack1ab: first order Jackknife, Jack2ab: second-order Jackknife, Chao 1: Chao estimator based on abundance and their bias-corrected complements (Jack1abp, Jack2abp, Chao1P).

		**Obs**	**S1**	**S2**	**Jack1ab**	**Jack1abp**	**Jack2ab**	**Jack2abp**	**Chao1**	**Chao1P**
**Candelaria**	**F1**	13	2	0	15	15.36	17	17.4	14	14.33
	**F2**	12	2	1	14	14.39	15	15.42	12.5	12.85
	**F3**	7	3	1	10	11.84	12	14.2	8.5	10.06
	**F4**	12	7	1	19	25.47	25	33.51	22.5	30.16
**Machay**	**F1**	13	3	3	16	16.85	16	16.85	13.75	14.48
	**F2**	11	7	1	18	25.29	24	33.72	21.5	30.21
	**F3**	14	7	2	21	26.25	26	32.5	21	26.25
	**F4**	12	1	1	13	13.09	13	13.09	12	12.08

**Table 6.** Average occupancy (ψ) and detection probabilities (p), based on the null models (ψ(.)p(.)) for the Andean bear, puma, oncilla and dogs in the Candelaria and Machay Reserves.

	**Delta AIC**	**ψ (± SE)**	***p* (± SE)**
**Andean bear**	4.59	0.6 (0.1)	0.27 (0.06)
**Puma**	3.34	0.4 (0.1)	0.17 (0.08)
**Oncilla**	0	0.33 (0.1)	0.1 (0.08)
**Domestic dogs**	0	0.2 (0.1)	0.22 (0.2)
