# Supplementary material for: First insights in terrestrial mammals monitoring in the Candelaria and Machay Reserves in the Ecuadorian Tropical Andes
Source: Biodivers Data J. 2023 Feb 27;11:e98119. doi: 10.3897/BDJ.11.e98119 (PMC10848741; doi:10.3897/BDJ.11.e98119)
Supplement: Supplementary material 1 — SM1 [file bdj-11-e98119-s001.docx]

| **Sampl** | **Btotal** | **Brepl** | **Brich** |
| --- | --- | --- | --- |
| Machay_F1 | 0.81450085 | 0.38242528 | 0.43207557 |
| Machay_F2 | 0.73965904 | 0.32580589 | 0.41385315 |
| Machay_F3 | 0.69011482 | 0.26521483 | 0.42489999 |
| Machay_F4 | 0.65323341 | 0.23397308 | 0.41926033 |
| Candelaria_F1 | 0.62226975 | 0.17275944 | 0.44951031 |
| Candelaria_F2 | 0.58451639 | 0.11567544 | 0.46884094 |
| Candelaria_F3 | 0.54399856 | 0.06565788 | 0.47834068 |
| Candelaria_F4 | 0.50344828 | 0 | 0.50344828 |

**Table SM1.** Table of accumulated beta diversity considering the beta diversity partitioning approach
