# Supplementary material for: First insights in terrestrial mammals monitoring in the Candelaria and Machay Reserves in the Ecuadorian Tropical Andes
Source: Biodivers Data J. 2023 Feb 27;11:e98119. doi: 10.3897/BDJ.11.e98119 (PMC10848741; doi:10.3897/BDJ.11.e98119)
Supplement: Supplementary material 2 — Detailed outcomes of the occupancy models assessed. [file bdj-11-e98119-s002.docx]

**SM2. Detailed outcomes of the occupancy models assessed.**

**Table 1.** Candidate models assessed for the Andean bear (*Tremarctos ornatus*) occupancy probability estimation. Changes in occupancy across sampling campaigns (i.e., colonization and extinction) were evaluated according to the rain seasonality and dog occurrence.

| Model | nPars | AIC | delta | AICwt | cumltvWt |
| --- | --- | --- | --- | --- | --- |
| ψ(Rdist)p(eff) | 8 | 449.12 | 0 | 0.7594 | 0.76 |
| ψ(.)p(.) | 4 | 453.71 | 4.59 | 0.0764 | 0.84 |
| ψ(NoVeg_bf)p(eff) | 8 | 454.13 | 5.01 | 0.0621 | 0.9 |
| ψ(Rugos)p(eff) | 8 | 456.74 | 7.62 | 0.0168 | 0.91 |
| ψ(Cdist)p(eff) | 8 | 456.85 | 7.73 | 0.0159 | 0.93 |
| ψ(Floss)p(eff) | 8 | 457.16 | 8.04 | 0.0136 | 0.94 |
| ψ(NoVeg_1x1)p(eff) | 8 | 457.49 | 8.37 | 0.0116 | 0.96 |
| ψ(NatVeg_bf)p(eff) | 8 | 458.03 | 8.91 | 0.0088 | 0.96 |
| ψ(PAdist)p(eff) | 8 | 458.83 | 9.71 | 0.0059 | 0.97 |
| ψ(NatVeg_1x1)p(eff) | 8 | 458.99 | 9.87 | 0.0055 | 0.98 |
| ψ(PastCrops_bf)p(eff) | 8 | 459.15 | 10.04 | 0.005 | 0.98 |
| ψ(Acces)p(eff) | 8 | 459.16 | 10.04 | 0.005 | 0.99 |
| ψ(EII)p(eff) | 8 | 459.27 | 10.15 | 0.0047 | 0.99 |
| ψ(PastCrops_1x1)p(eff) | 8 | 459.32 | 10.2 | 0.0046 | 1 |
| ψ(Fgain)p(eff) | 8 | 459.33 | 10.22 | 0.0046 | 1 |

ψ: Occupancy probability; p: detection probability.


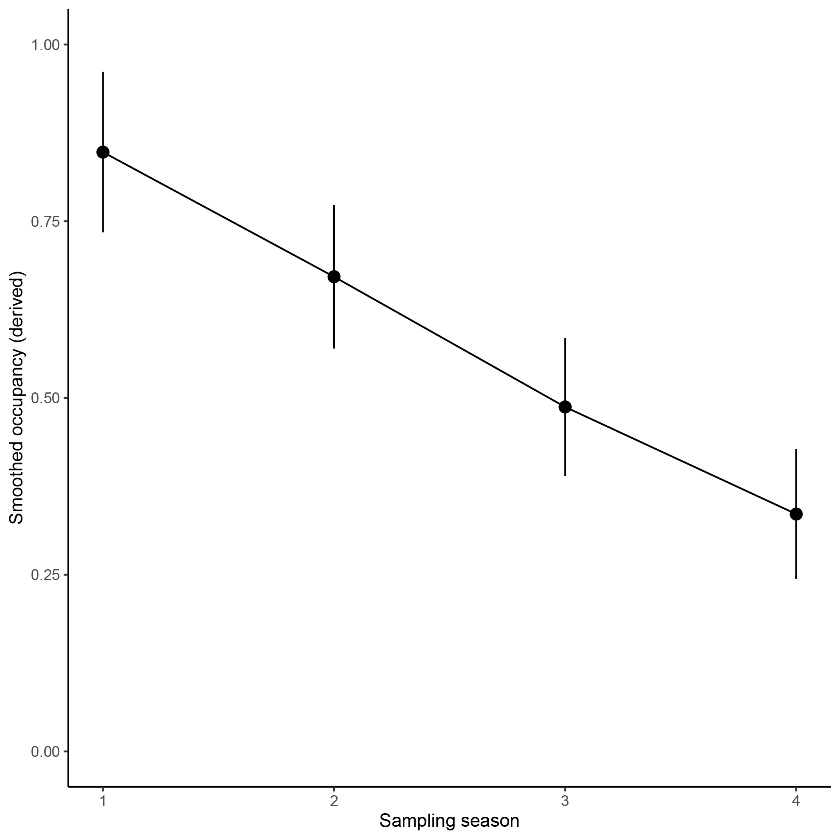


**Figure 1.** Smoothed null occupancy probability estimation for the Andean bear (*Tremarctos ornatus*) across sampling campaigns.

**Table 2.** Candidate models assessed for the oncilla (*Leopardus tigrinus*) occupancy probability estimation. Changes in occupancy across sampling campaigns (i.e., colonization and extinction) were evaluated according to the rain seasonality and dog occurrence.

| Model | nPars | AIC | delta | AICwt | cumltvWt |
| --- | --- | --- | --- | --- | --- |
| ψ(.)p(.) | 4 | 189.19 | 0 | 0.294 | 0.29 |
| ψ(Fgain)p(eff) | 8 | 189.55 | 0.36 | 0.245 | 0.54 |
| ψ(Acces)p(eff) | 8 | 192.47 | 3.29 | 0.057 | 0.59 |
| ψ(NoVeg_1x1)p(eff) | 8 | 192.61 | 3.43 | 0.053 | 0.65 |
| ψ(PastCrops_1x1)p(eff) | 8 | 193.02 | 3.84 | 0.043 | 0.69 |
| ψ(PastCrops_bf)p(eff) | 8 | 193.21 | 4.03 | 0.039 | 0.73 |
| ψ(NoVeg_bf)p(eff) | 8 | 193.32 | 4.13 | 0.037 | 0.77 |
| ψ(Rdist)p(eff) | 8 | 193.41 | 4.23 | 0.035 | 0.8 |
| ψ(Floss)p(eff) | 8 | 193.47 | 4.28 | 0.034 | 0.84 |
| ψ(NatVeg_bf)p(eff) | 8 | 193.59 | 4.4 | 0.032 | 0.87 |
| ψ(NatVeg_1x1)p(eff) | 8 | 193.7 | 4.52 | 0.031 | 0.9 |
| ψ(EII)p(eff) | 8 | 193.71 | 4.53 | 0.031 | 0.93 |
| ψ(Rugos)p(eff) | 8 | 194.09 | 4.91 | 0.025 | 0.96 |
| ψ(PAdist)p(eff) | 8 | 194.35 | 5.16 | 0.022 | 0.98 |
| ψ(Cdist)p(eff) | 8 | 194.4 | 5.21 | 0.022 | 1 |

ψ: Occupancy probability; p: detection probability.


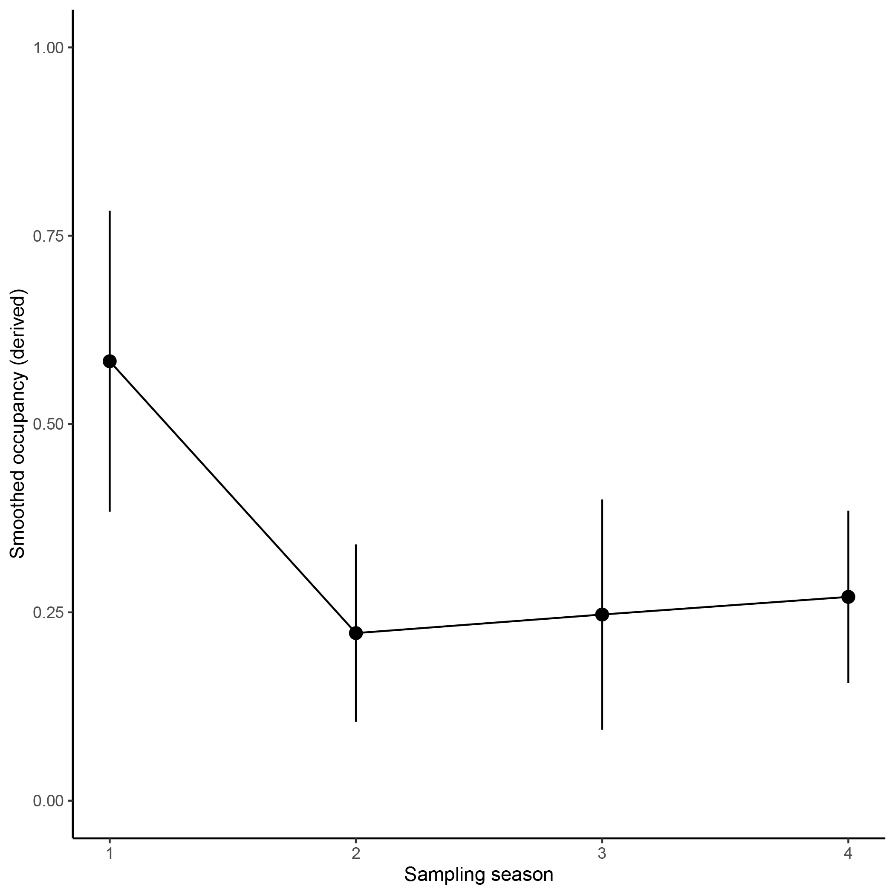


**Figure 2.** Smoothed null occupancy probability estimation for the oncilla (*Leopardus tigrinus*) across sampling campaigns.

**Table 3.** Candidate models assessed for the puma (*Puma concolor*) occupancy probability estimation. Changes in occupancy across sampling campaigns (i.e., colonization and extinction) were evaluated according to the rain seasonality and dog occurrence.

| Model | nPars | AIC | delta | AICwt | cumltvWt |
| --- | --- | --- | --- | --- | --- |
| ψ(NoVeg_bf)p(eff) | 8 | 257.24 | 0 | 0.392 | 0.39 |
| ψ(Cdist)p(eff) | 8 | 259.06 | 1.81 | 0.158 | 0.55 |
| ψ(.)p(.) | 4 | 260.58 | 3.34 | 0.074 | 0.62 |
| ψ(Rdist)p(eff) | 8 | 261.28 | 4.04 | 0.052 | 0.68 |
| ψ(NatVeg_1x1)p(eff) | 8 | 261.42 | 4.18 | 0.049 | 0.73 |
| ψ(Acces)p(eff) | 8 | 262.07 | 4.83 | 0.035 | 0.76 |
| ψ(PastCrops_1x1)p(eff) | 8 | 262.09 | 4.85 | 0.035 | 0.79 |
| ψ(NatVeg_bf)p(eff) | 8 | 262.1 | 4.86 | 0.035 | 0.83 |
| ψ(Fgain)p(eff) | 8 | 262.34 | 5.1 | 0.031 | 0.86 |
| ψ(PAdist)p(eff) | 8 | 262.7 | 5.46 | 0.026 | 0.89 |
| ψ(PastCrops_bf)p(eff) | 8 | 262.75 | 5.51 | 0.025 | 0.91 |
| ψ(Floss)p(eff) | 8 | 262.84 | 5.6 | 0.024 | 0.93 |
| ψ(EII)p(eff) | 8 | 262.86 | 5.62 | 0.024 | 0.96 |
| ψ(Rugos)p(eff) | 8 | 263.07 | 5.83 | 0.021 | 0.98 |
| ψ(NoVeg_1x1)p(eff) | 8 | 263.15 | 5.91 | 0.02 | 1 |

ψ: Occupancy probability; p: detection probability.


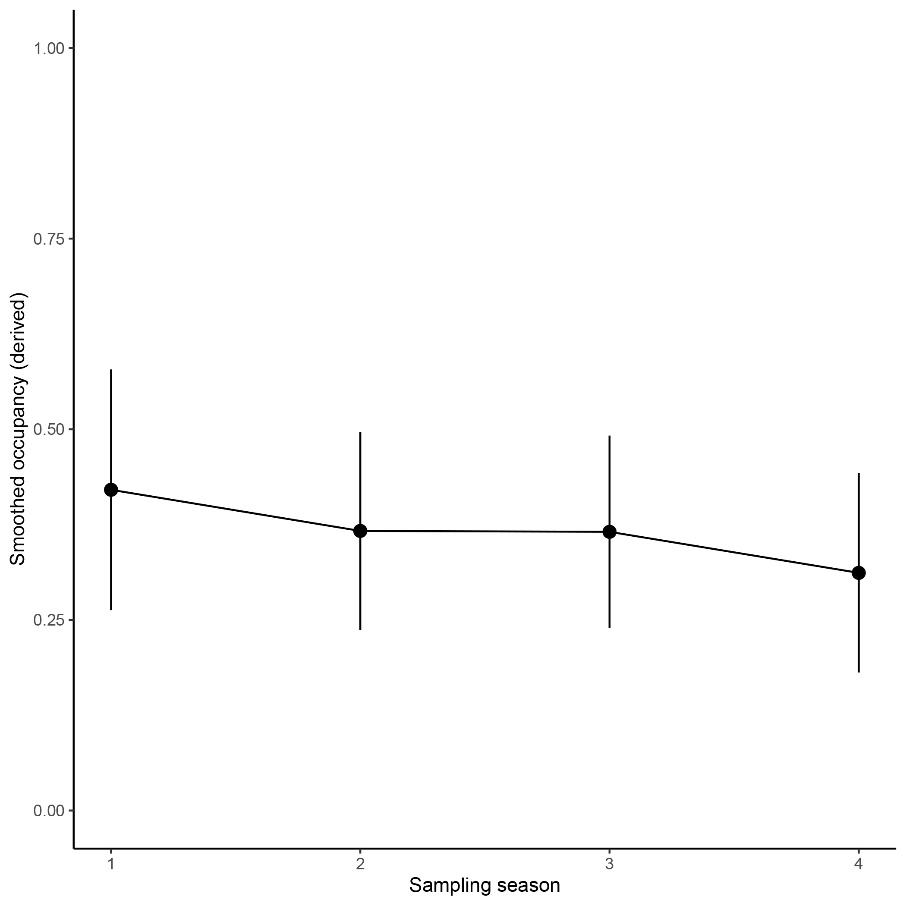


**Figure 3.** Smoothed null occupancy probability estimation for the puma (*Puma concolor*) across sampling campaigns.

**Table 4.** Candidate models assessed for domestic dogs (*Canis familiaris*) occupancy probability estimation. Changes in occupancy across sampling campaigns (i.e., colonization and extinction) were evaluated according to the rain seasonality and dog occurrence.

| Model | nPars | AIC | delta | AICwt | cumltvWt |
| --- | --- | --- | --- | --- | --- |
| ψ(.)p(.) | 4 | 198.24 | 0 | 0.342 | 0.34 |
| ψ(Fgain)p(eff) | 8 | 199.45 | 1.21 | 0.187 | 0.53 |
| ψ(NatVeg_1x1)p(eff) | 8 | 201.25 | 3.02 | 0.076 | 0.6 |
| ψ(Rdist)p(eff) | 8 | 201.37 | 3.13 | 0.072 | 0.68 |
| ψ(Acces)p(eff) | 8 | 201.76 | 3.52 | 0.059 | 0.74 |
| ψ(PAdist)p(eff) | 8 | 202.75 | 4.51 | 0.036 | 0.77 |
| ψ(NoVeg_1x1)p(eff) | 8 | 202.77 | 4.53 | 0.036 | 0.81 |
| ψ(Floss)p(eff) | 8 | 203.06 | 4.82 | 0.031 | 0.84 |
| ψ(PastCrops_bf)p(eff) | 8 | 203.35 | 5.12 | 0.027 | 0.86 |
| ψ(PastCrops_1x1)p(eff) | 8 | 203.4 | 5.16 | 0.026 | 0.89 |
| ψ(Cdist)p(eff) | 8 | 203.62 | 5.38 | 0.023 | 0.91 |
| ψ(NatVeg_bf)p(eff) | 8 | 203.64 | 5.4 | 0.023 | 0.94 |
| ψ(NoVeg_bf)p(eff) | 8 | 203.68 | 5.44 | 0.023 | 0.96 |
| ψ(Rugos)p(eff) | 8 | 203.83 | 5.6 | 0.021 | 0.98 |
| ψ(EII)p(eff) | 8 | 203.84 | 5.6 | 0.021 | 1 |

ψ: Occupancy probability; p: detection probability.


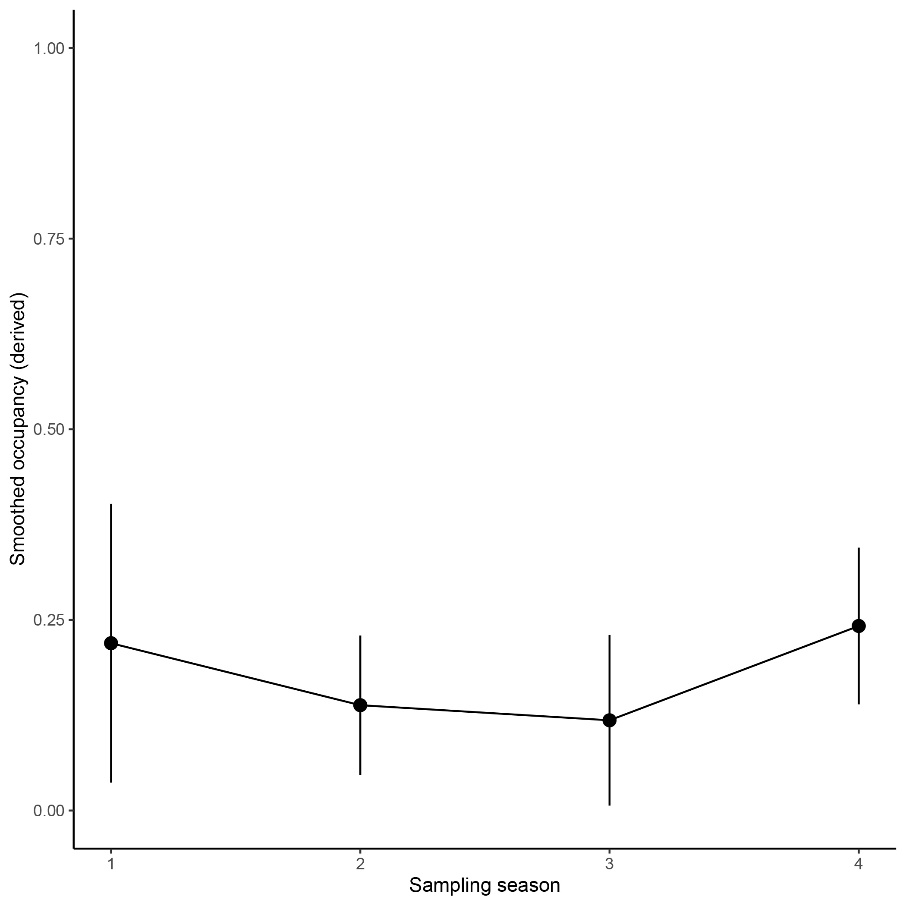


**Figure 4.** Smoothed null occupancy probability estimation for the domestic dog (*Canis familiaris*) across sampling campaigns.
